# Lower Vitamin D Status Is Associated with an Increased Risk of Ischemic Stroke: A Systematic Review and Meta-Analysis

**DOI:** 10.3390/nu10030277

**Published:** 2018-02-28

**Authors:** Ren Zhou, Mengying Wang, Hui Huang, Wenyong Li, Yonghua Hu, Tao Wu

**Affiliations:** 1 School of Public Health, Peking University Health Science Center, Beijing 100191, China; rzhou@bjmu.edu.cn (R.Z.); mywang@bjmu.edu.cn (M.W.); hh_1110306110@bjmu.edu.cn (H.H.); lwy0000@bjmu.edu.cn (W.L.); yhhu@bjmu.edu.cn (Y.H.); 2 Key Laboratory of Reproductive Health, Ministry of Health, Beijing 100191, China

**Keywords:** vitamin D, stroke, systematic review, meta-analysis

## Abstract

In recent years, accumulating evidence has supported the hypothesis that lower vitamin D status is associated with several known risk factors of stroke. However, the relationship between vitamin D and stroke is still uncertain. To explore if there was an association between vitamin D status and the risk of stroke, a systematic review and a meta-analysis were conducted by searching three databases: Pubmed, Embase, and the Cochrane Library. Following the application of inclusion and exclusion criteria, the relative risk estimates of all the included studies were pooled together to compare the risk of stroke between the lowest and the highest category of vitamin D. The Newcastle–Ottawa Scale (NOS) and the Cochrane Risk of Bias Tool were used to assess the risk of bias, and the publication bias was detected by using a funnel plot and Egger’s test. Nineteen studies were included and the pooled relative risk was 1.62 (95% CI: 1.34–1.96). Further analysis found that vitamin D status was associated with ischemic stroke (relative risk = 2.45, 95% CI: 1.56–3.86), but not with hemorrhagic stroke (relative risk = 2.50, 95% CI: 0.87–7.15). In conclusion, our meta-analysis supported the hypothesis that lower vitamin D status was associated with an increased risk of ischemic stroke. Further studies are required to confirm this association and to explore the association among different subtypes.

## 1. Introduction

Stroke is the second leading cause of death among people aged 60 years and above [1], and is causing a substantial global disease burden. Based on the global burden disease (GBD) 2013 study, about 6.5 million people died from stroke and 10.3 million people suffered a stroke in 2013 [2]. The situation was even more serious in China: according to a nationwide population-based survey conducted in 2013, China had about 2.4 million incidences of stroke each year and bore the heaviest burden of stroke across the world [3]. Although hypertension, diabetes, obesity, and tobacco use have been ascertained as important risk factors of stroke, the exploration of unknown risk factors is still ongoing, especially for those factors that are easily to be intervened. 

Vitamin D, which is classically known as a protective factor in bone metabolism, in recent years has been reported to play a vital role in cardiovascular health [4,5,6]. An increased incidence of stroke was found among older women whose serum 25(OH)D concentration was less than 50 nmol/L [7]. Some evidence also supported the hypothesis that low vitamin D intake may function as a predictor of long-term incidence of stroke [8,9]. Moreover, several meta-analyses published respectively in 2012 and 2013 [10,11], also yielded significant results which show that low vitamin D status was associated with a higher risk of stroke. However, these meta-analyses only focused on the circulating vitamin D when exploring the association between vitamin D and the risk of stroke. Vitamin intake is also a reliable indicator of vitamin D status in the body. Further, instead of targeting stroke, Brøndum’s meta-analysis [10] only focused on the ischemic stroke, and Wang’s study [11] used composite end points, including cardiovascular disease (CVD) death, myocardial infarction, and stroke. 

In addition, a recent study that combined the data of three population-based studies reported no significant association between vitamin D status and the incidence of hypertension and stroke [12,13]. Moreover, another study using Mendelian randomization did not support the claim that low vitamin D status had a causal relationship with ischemic stroke [14]. Accordingly, the relationship between vitamin D and the risk of stroke was still inconclusive, and efforts were needed to verify the association between vitamin D and the incidence of stroke. Consequently, a systematic review and meta-analysis was performed to better understand if vitamin D status potentially influenced the risk of stroke.

## 2. Methods 

This systematic review and meta-analysis was based on a protocol published in the 2017 PROSPERO register (CRD: 42017076416), and the report followed the PRISMA (Preferred Reporting Items for Systematic Reviews and Meta-Analyses) guidelines.

### 2.1. Search Strategy

A literature search was performed using three databases: Pubmed, Embase, and the Cochrane Library, from inception through to 30 September 2017. When searching, Medical Subject Headings (MeSH) terms and free text words were combined. Detailed search strategies for each database are shown in Table 1.

### 2.2. Inclusion and Exclusion Criteria

The inclusion criteria were as follows:

1. Published in English.
2. Conducted among humans.
3. Being a case control study, a cohort study, or a randomized controlled trial (RCT).
4. Studying the association between vitamin D status (including indicators such as circulating vitamin D, and vitamin D intake) and the risk of incident stroke.
5. Containing information about contingency tables, the odds ratio (OR), or the risk ratio (RR), as well as their 95% confidence interval (CI).

The exclusion criteria were as follows:

1. Repeating publications or duplicates.
2. Insufficient data for analysis.

### 2.3. Study Selection and Data Extraction

Two reviewers independently completed the study selection and data extraction. Disagreements in the study selection and data extraction process were resolved by discussion. The study selection was conducted according to a strict adherence to the inclusion and exclusion criteria. If several studies used the same data source for analysis, the one with the largest sample size was included and the others were excluded. As for the data extraction, information including the first author, publication year, journal, country or district, study design, sample size, OR or RR and their 95% CI, adjusted variables, different subtypes of stroke, and the vitamin D status were extracted. If several models were reported, the estimates in the most adjusted models were extracted.

### 2.4. Risk of Bias Assessment

Two different assessment tools were used for different study designs. The Newcastle–Ottawa Scale (NOS) was used to assess the quality of non-randomized studies, including case control studies and cohort studies. The scale includes eight items with one or two points for each item. Studies with zero to three points were considered with a high risk of bias. As for the RCTs, the Cochrane Risk of Bias Tool with eight items was used to assess the risk of bias, with one point for each item. Studies with zero to two points were considered with a high risk of bias.

### 2.5. Data Analysis and Statistical Methods

Risk ratios between the highest and the lowest category of vitamin D in prospective studies and the odds ratios in retrospective studies were used to perform the data synthesis. The pooled relative risk and the 95% CI of the included studies were calculated using the inverse variance method, with ORs of retrospective studies being approximately considered as RRs. *Q* test and *I*^2^ statistics were used to assess the heterogeneity among the included studies. If no significant heterogeneity existed (*I*^2^ statistics less than 50%), the pooled estimate and the 95% CI were calculated using a fixed-effect model. If significant heterogeneity was present (*I*^2^ statistics no less than 50%), then a random-effect model was adopted. Review Manager 5.3 (The Cochrane Collaboration, Copenhagen, Denmark) was used for data analyses.

### 2.6. Sensitivity Analyses

Two kinds of sensitivity analyses were performed. Studies with extraordinary estimates of the association (vitamin D and stroke), and studies with scores of less than 7 in the assessment of risk of bias were respectively removed. After the removal, the pooled relative risk was calculated again using the remaining studies. If the pooled estimates changed from significant to insignificant (or vice versa), or the pooled estimates changed from more than 1 to no more than 1 (or vice versa), we then considered that influential outliers existed among the included studies.

### 2.7. Subgroup Analysis

Further subgroup analyses were also performed according to study designs (case control study, prospective studies), sample size (more or no more than 1000), subtypes of stroke (ischemic stroke or hemorrhagic stroke), and vitamin D indicators (circulating vitamin D or vitamin D intake), to explore if the association differs in different subgroups and to locate the origin of the heterogeneity.

### 2.8. Publication Bias

A funnel plot was used to visually assess the publication bias. Egger’s test (significant level = 0.05) was conducted to quantitatively explore the possible publication bias, using STATA 14.0 (StataCorp LP, College Station, TX, USA).

## 3. Results

The meta-analysis and report of the results were based on the PRISMA checklist and the details are shown in Table S1. This report included all of the items in the PRISMA checklist. 

### 3.1. Characteristics of Included Studies

**Figure 1:**
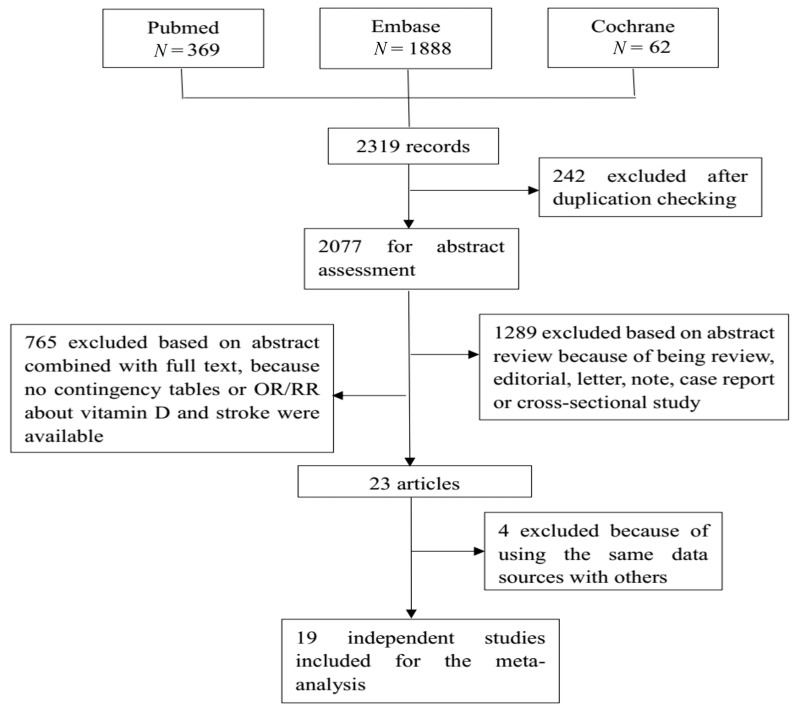
This is the flow chart of the selection of studies eligible for our meta-analysis. A total of 2319 records were retrieved from three databases: Pubmed, Embase, and the Cochrane library. After a strict selection process based on the inclusion and exclusion criteria, 19 studies were eligible for the meta-analysis.

A total of 2319 studies were retrieved from the three databases, of which 19 studies were finally included for analysis. Figure 1 shows the flow diagram. There were three case control studies and 16 prospective studies, including the cohort study, and the randomized controlled trial (RCT). Seven studies contained subtype information of stroke, with seven for ischemic stroke and four for hemorrhagic stroke. Three studies focused on the effect of vitamin D intake on stroke, while seventeen studies used the circulating vitamin D as an indicator. Table 2 shows detailed information of each of the included studies, incorporating countries or districts, sample size, event of interest, number of cases, study design, and adjusted odds ratios (ORs) or risk ratios (RRs) and their 95% confidence interval (CI). Most studies provided estimates that had been adjusted by sex, age, and some other key confounders. Table S3 shows the adjusted variables in each original study. 

### 3.2. Overall Analysis

**Figure 2:**
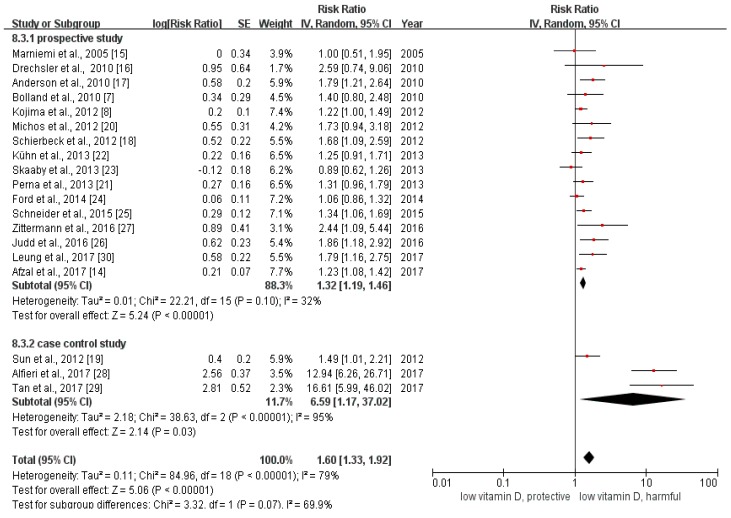
This is the forest plot for the pooled analysis of all 19 included studies. The overall analysis was executed by Review Manager 5.3, using the random-effect model.

We calculated the relative risk between the lowest with the highest categories of vitamin D in all of the included studies. The pooled relative risk was 1.60 (95% CI: 1.33–1.92, Figure 2). The *p* value of the *Q* test was less than 0.01 and the *I*^2^ statistic = 79%, indicating that heterogeneity existed among the included studies.

### 3.3. Subgroup Analysis

The results of the subgroup analysis were summarized in Table S2. Grouping the total 19 studies by study design, the pooled odds ratio between the lowest with the highest categories of vitamin D of the three case control studies was 6.59 (95% CI: 1.17–37.02), while the pooled estimate of RR among the 16 prospective studies (including 15 cohort studies and one RCT) was 1.28 (95% CI: 1.19–1.38). The estimated relative risk of large sample studies (sample size > 1000) was 1.28 (95% CI: 1.18–1.38), and that of the small sample studies (sample size ≤ 1000) was 3.63 (95% CI: 1.30–10.15). The pooled relative risk of ischemic stroke was 2.36 (95% CI: 1.53–3.63), which was similar to the overall estimate of RR. We found no association between vitamin D status and hemorrhagic stroke (RR = 2.50, 95% CI: 0.87–7.15). The pooled relative risk for those studies using circulating vitamin D as an indicator was 1.93 (95% CI: 1.49–2.48). As for the studies reporting the information about vitamin D intake and stroke, the pooled relative risk was statistically insignificant (RR = 1.22, 95% CI: 0.96–1.55).

### 3.4. Risk of Bias Assessment

The assessment of risk of quality showed that most of the included studies had a low risk of bias, except for one study with a medium risk of bias (details shown in Table 3).

### 3.5. Sensitivity Analysis

After removing the two studies [28,29] with the strongest estimates of the association, the pooled relative risk for the remaining studies was 1.32 (95% CI: 1.19–1.46). After removing the studies with scores of less than seven in the assessment of risk of bias [27], the estimate was 1.58 (95% CI: 1.31–1.89). In addition, the effect estimates from Tan et al. [29] were based on a model that did not control any covariates. After removing the study, the pooled estimate did not change substantially. The sensitivity analyses identified no influential outlier.

### 3.6. Publication Bias

**Figure 3:**
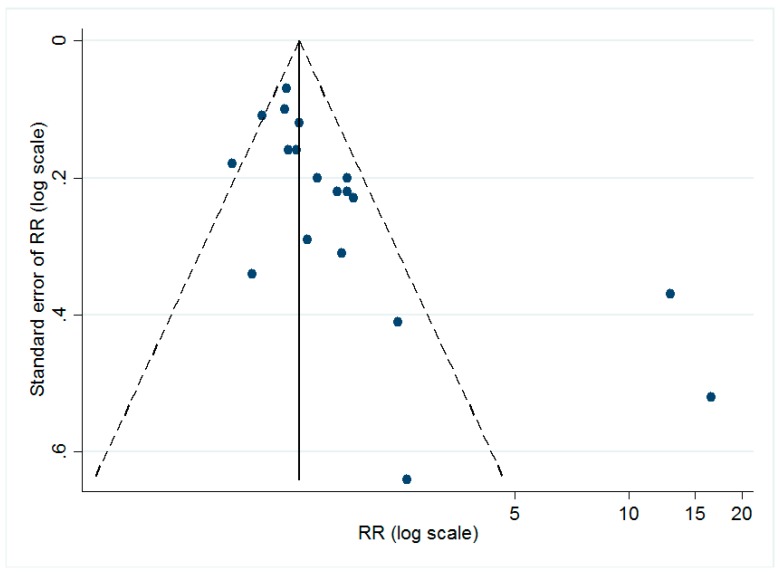
Funnel plot for all of the included studies. As shown, the dots, which represented each individual study, are distributed asymmetrically, indicating the possible existence of publication bias.

The dissymmetry of the funnel plot (shown in Figure 3) implied the possible existence of publication bias. The Egger’s test (*t* = 3.32, *p* < 0.05) also suggested that there might be small-study effects in our study.

## 4. Discussion

Our meta-analysis included 19 studies that explored the association of vitamin D status with the risk of stroke. Comparing the lowest and highest category of vitamin D using the random-effect model, the pooled relative risk for all included studies was 1.62 [95% of confidence interval (CI): 1.34–1.96], which indicated an inverse association between vitamin D status and the risk of stroke. Further subgroup analyses were performed and found that study designs and sample size did not affect the association between vitamin D status and the risk of stroke. The subgroup analyses indicated that vitamin D status was associated with ischemic stroke, but not with hemorrhagic stroke. In addition, we did not find a significant association between vitamin D intake and stroke. 

Our results were generally consistent with two earlier meta-analyses. Wang et al. [11] reported that the pooled risk ratio (RR) was 1.64 (95% CI: 1.27–2.10), and Brøndum et al. [10] found a similar result for the effect of vitamin D on ischemic stroke [pooled odds ratio (OR) = 1.67, 95% CI: 1.43–1.96]. The consistent results of the meta-analyses indicated that low vitamin D status was a possible risk factor of stroke. 

Many studies have provided evidence for possible mechanisms to explain the effect of vitamin D on stroke. Although vitamin D is known for its regulation of bone health, vitamin D receptor (VDR) was expressed in most human tissues and cells [31]. More importantly, VDR also exists in the vascular smooth muscle cell [32], the platelet [33], and many other immune cells [34]. Since these cells play important roles in the development of stroke, they may be a possible mechanism that links vitamin D and stroke. Experiments on animals also observed that vitamin D could inhibit thrombosis [35], which could be supporting evidence to explain why low vitamin D status increases the risk of ischemic stroke. In addition, low vitamin D status has been associated with the up-regulation of the renin–angiotensin system (RAS), both in experimental mice [36] and in healthy humans [37]. RAS is a vital pathway in the regulation of the cardiovascular system. Thus, the regulation of the RAS may be another possible mechanism through which vitamin D affects the risk of stroke. Inflammation, which could drive the progress of cardiovascular diseases [38,39], could also be regulated by vitamin D. Vitamin D could inhibit the production of inflammation factors, such as interleukin 6 (IL-6) and tumor necrosis factor alpha (TNF-α) [40], and as a result affect the progress of stroke.

In the subgroup analyses, the estimated effect size of the case control studies showed a stronger association between vitamin D status and stroke than that of the prospective studies, which might be mainly due to the two case control studies with distinct and higher estimated ORs [28,29]. Since these two studies had a low risk of bias according to the risk of bias assessment, the results may be due to the especially small sample size (less than 500).

Our study did not support the hypothesis that vitamin D status had an effect on hemorrhagic stroke. As mentioned above, vitamin D can inhibit the development of thrombosis, which may provide a rational explanation for the relationship between vitamin D and ischemic stroke. However, this explanation was not suitable for hemorrhagic stroke as hemorrhagic stroke was not based on the development of thrombosis. Nevertheless, vitamin D might induce hemorrhagic stroke through other mechanisms, such as inflammation and the RAS. Since the number of original studies exploring the association between vitamin D status and stroke was quite limited, more studies are needed to further investigate the association between vitamin D and the risk of hemorrhagic stroke. 

The estimated relative risk in the vitamin D intake group was insignificant. Firstly, only three studies that reported the vitamin D intake were included in our analysis, resulting in the limited power to identify the association. In addition, the measurement of vitamin D intake might differ among different studies. Further studies on the intake of vitamin D will provide better evidence for estimating.

Recently, a Mendelian randomized study was conducted to explore the causal relationship between serum vitamin D and the risk of ischemic stroke [14]. Although the study did not support that serum vitamin D was a causal factor for ischemic stroke, the authors did declare that only odds ratios of more than 1.5 could be found with 80% power. Insufficient power could lead to a false negative association. Thus, the causal relationship between vitamin D and ischemic stroke could not be excluded. The Mendelian randomized study provides a valid approach to explore the causal relationship between possible risk factors and diseases, by controlling multiple environmental confounders. However, extremely large sample sizes are necessary to achieve positive results. If genetic variation only explains a small proportion of the variance of the exposures being studied [41], this limits the power of a Mendelian randomized study.

In the current study, we updated older meta-analyses using recently published studies. In addition, to explore the heterogeneity among these studies, further subgroup analyses were conducted. This study found that vitamin D might play different roles (or no role at all) in different subtypes. However, our study has several limitations. Firstly, we estimated the association between vitamin D status and stroke by comparing the lowest and highest category of vitamin D. However, the category standards were not the same among our included studies. This heterogeneity among studies might introduce unknown influence on the pooled estimates. To resolve this problem, when the heterogeneity was high, the random-effect model was used to calculate the pooled estimate. Second, the funnel plot and Egger’s test indicated that publication bias might exist in our studies. Since this study used published studies as data sources, it was difficult to prevent publication bias. Thirdly, as the original studies included different confounder sets in the fully adjusted models, heterogeneity could be introduced into the meta-analysis. However, a previous study reported that choosing effect estimates based on the fully adjusted models would not substantially change the results of the meta-analysis, compared to when using models that control only the most common confounders (such as sex and age) [42]. Therefore, the heterogeneity of different confounder sets among the original studies would not substantially influence the results. 

## 5. Conclusions

Lower vitamin D status was associated with an increased risk of stroke by synthesizing the data of nineteen relevant studies. This association remained in the ischemic stroke group, while the relationship between vitamin D status and hemorrhagic stroke was insignificant. Higher circulating vitamin D was a significant protective factor for stroke, while the association between vitamin D intake and the risk of stroke remained to be further explored. More studies, especially randomized controlled trials, are needed to verify this association and explore the prevention effectiveness of vitamin D supplement.

## Figures and Tables

**Table 1:** Search strategies and results.

Database	Search Strategy	Retrieved Records
Pubmed	((stroke[MeSH Terms]) OR (cerebrovascular accident) OR (CVA) OR (brain vascular accident) OR (cerebrovascular stroke) OR (cerebral stroke)) AND ((vitamin D[MeSH Terms]) OR (ergocalciferol) OR (25(OH)D) OR (25-hydroxyvitamin D) OR (cholecalciferol))	369
Embase	(‘cerebrovascular accident’/exp OR ‘stroke’ OR ‘CVA’ OR ‘brain ischemia’ OR ‘brain vascular accident’ OR ‘cerebrovascular stroke’ OR ‘cerebral stroke’) AND (‘vitamin D’/exp OR ‘ergocalciferol’ OR ‘25(OH)D’ OR ‘25-hydroxyvitamin D’ OR ‘cholecalciferol’) AND [english]/lim AND [embase]/lim	1888
Cochrane library	((stroke[MeSH Terms]) OR (cerebrovascular accident) OR (CVA) OR (brain vascular accident) OR (cerebrovascular stroke) OR (cerebral stroke)) AND ((vitamin D[MeSH Terms]) OR (ergocalciferol) OR (25(OH)D) OR (25-hydroxyvitamin D) OR (cholecalciferol))	62

**Table 2:** Estimates of association between vitamin D status and the risk of stroke in 19 studies included in the meta-analysis.

Study	Countries/Districts	Sample Size	Event	Number of Cases	Study Design	OR/RR 95% CI
Marniemi et al., 2005 [15]	Finland	755	stroke	70	cohort study	1.00 (0.52, 1.96), for serum 25(OH)D
						2.20 (1.11, 4.35), for vitamin intake
Bolland et al., 2010 [7]	New Zealand	1471	stroke	59	cohort study	1.4 (0.8, 2.5)
Drechsler et al., 2010 [16]	Germany	1108	stroke	89	cohort study	2.58 (0.74, 8.98)
Anderson et al., 2010 [17]	The US	26,025	stroke	208	cohort study	1.78 (1.2, 2.66)
Schierbeck et al., 2012 [18]	Denmark	2013	stroke	89	cohort study	1.68 (1.10, 2.56)
Kojima et al., 2012 [8]	Hawaii	7385	stroke	960	cohort study	1.22 (1.01, 1.47)
			ischemic stroke	651		1.27 (1.01, 1.59)
			hemorrhagic stroke	269		0.97 (0.68, 1.38)
			unknown	40		
Sun et al., 2012 [19]	The US	928	ischemic stroke	464	case control study	1.49 (1.01, 2.18)
Michos et al., 2012 [20]	The US	7981	fatal stroke	176	cohort study	1.74 (0.94, 3.2)
Perna et al., 2013 [21]	Germany	7709	stroke	353	cohort study	1.31 (0.95, 1.81)
Kühn et al., 2013 [22]	Germany	2603	stroke	471	cohort study	1.25 (0.92, 1.70)
Skaaby et al., 2013 [23]	Denmark	8131	stroke	316	cohort study	0.88 (0.63, 1.25)
Ford et al., 2014 [24]	The UK	5292	stroke	309	RCT	1.06 (0.85, 1.32)
Schneider et al., 2015 [25]	The US	12,158	stroke	804	cohort study	1.34 (1.06, 1.71)
Judd et al., 2016 [26]	The US	1547	stroke	610	cohort study	1.85 (1.17, 2.93)
			ischemic stroke	536		1.84 (1.14, 2.97)
			hemorrhagic stroke	74		1.82 (0.91, 3.65)
Zittermann et al., 2016 [27]	Germany	154	stroke	27	cohort study	2.44 (1.09, 5.45)
			ischemic stroke	13		2.36 (0.78, 7.19)
			hemorrhagic stroke	14		1.91 (0.67, 5.46)
Alfieri et al., 2017 [28]	Brazil	286	ischemic stroke	168	case control study	16.64 (5.66, 42.92)
Tan et al., 2017 [29]	China	404	stroke	224	case control study	12.92 (6.23, 26.82)
			ischemic stroke	121		11.67 (4.82, 28.27)
			hemorrhagic stroke	103		14.67 (5.38, 40.02)
Leung et al., 2017 [30]	Hong Kong	3458	stroke	244	cohort study	1.78 (1.16, 2.74)
Afzal et al., 2017 [14]	Denmark	35,152	ischemic stroke	1660	cohort study	1.23 (1.06, 1.42)

OR, odds ratio; RR, risk ratio, the ORs or RRs have been adjusted by key confounders in the original studies.

**Table 3:** The results of the risk of bias assessment.

Study	Selection of the Study Groups	Comparability of the Groups	Ascertainment of the Exposure or Outcome	Total Score	Risk of Bias
**Case control study ^1^**					
Sun et al., 2012 [19]	4	2	3	9	low
Alfieri et al., 2017 [28]	4	2	3	9	low
Tan et al., 2017 [29]	2	2	3	7	low
**Cohort study ^1^**					
Marniemi et al., 2005 [15]	4	1	3	8	low
Bolland et al., 2010 [7]	4	2	3	9	low
Anderson et al., 2010 [17]	4	2	3	9	low
Drechsler et al., 2010 [16]	3	2	3	8	low
Schierbeck et al., 2012 [18]	3	2	3	8	low
Kojima et al., 2012 [8]	4	2	3	9	low
Michos et al., 2012 [20]	4	1	3	8	low
Judd et al., 2016 [26]	4	2	3	9	low
Perna et al., 2013 [21]	4	2	3	9	low
Kühn et al., 2013 [22]	4	2	3	9	low
Skaaby et al., 2013 [23]	3	1	3	7	low
Schneider et al., 2015 [25]	4	1	3	8	low
Zittermann et al., 2016 [27]	3	1	2	6	medium
Leung et al., 2017 [30]	3	1	3	7	low
Afzal et al., 2017 [14]	4	2	3	9	low
**RCT ^2^**					
Ford et al., 2014 [24]		6	low		

^1^ The assessment for case control studies and cohort studies was conducted using the Newcastle–Ottawa Scale. ^2^ RCT means randomized controlled trials. The assessment for RCTs and cohort studies was conducted using the Cochrane Risk of Bias Tool.

## Supplementary Materials

The following are available online at http://www.mdpi.com/2072-6643/10/3/277/s1, Table S1: The details in this review of items in PRISMA guidelines, Table S2: The results of subgroup analysis, Table S3: Variables adjusted in the model of each included study.
