# Supplementary material for: Lower Vitamin D Status Is Associated with an Increased Risk of Ischemic Stroke: A Systematic Review and Meta-Analysis
Source: Nutrients. 2018 Feb 28;10(3):277. doi: 10.3390/nu10030277 (PMC5872695; doi:10.3390/nu10030277)
Supplement: Supplementary file 1 [file nutrients-10-00277-s001.pdf]

Table S1. PRISMA Checklist

Section/topic	#	Checklist item	Reported on page #
**TITLE**			
Title	1	Identify the report as a systematic review, meta-analysis, or both.	1
**ABSTRACT**			
Structured summary	2	Provide a structured summary including, as applicable: background; objectives; data sources; study eligibility criteria, participants, and interventions; study appraisal and synthesis methods; results; limitations; conclusions and implications of key findings; systematic review registration number.	1
**INTRODUCTION**			
Rationale	3	Describe the rationale for the review in the context of what is already known.	1-2
Objectives	4	Provide an explicit statement of questions being addressed with reference to participants, interventions, comparisons, outcomes, and study design (PICOS).	2
**METHODS**			
Protocol and registration	5	Indicate if a review protocol exists, if and where it can be accessed (e.g., Web address), and, if available, provide registration information including registration number.	2
Eligibility criteria	6	Specify study characteristics (e.g., PICOS, length of follow-up) and report characteristics (e.g., years considered, language, publication status) used as criteria for eligibility, giving rationale.	2-3
Information sources	7	Describe all information sources (e.g., databases with dates of coverage, contact with study authors to identify additional studies) in the search and date last searched.	2
Search	8	Present full electronic search strategy for at least one database, including any limits used, such that it could be repeated.	2
Study selection	9	State the process for selecting studies (i.e., screening, eligibility, included in systematic review, and, if applicable, included in the meta-analysis).	3
Data collection process	10	Describe method of data extraction from reports (e.g., piloted forms, independently, in duplicate) and any processes for obtaining and confirming data from investigators.	3
Data items	11	List and define all variables for which data were sought (e.g., PICOS, funding sources) and any assumptions and simplifications made.	3
Risk of bias in individual studies	12	Describe methods used for assessing risk of bias of individual studies (including specification of whether this was done at the study or outcome level), and how this information is to be used in any data synthesis.	3
Summary measures	13	State the principal summary measures (e.g., risk ratio, difference in means).	3
Synthesis of results	14	Describe the methods of handling data and combining results of studies, if done, including measures of consistency (e.g., I^2) for each meta-analysis.	3

Table S1. PRISMA Checklist

Section/topic	#	Checklist item	Reported on page #
Risk of bias across studies	15	Specify any assessment of risk of bias that may affect the cumulative evidence (e.g., publication bias, selective reporting within studies).	4
Additional analyses	16	Describe methods of additional analyses (e.g., sensitivity or subgroup analyses, meta-regression), if done, indicating which were pre-specified.	3
**RESULTS**			
Study selection	17	Give numbers of studies screened, assessed for eligibility, and included in the review, with reasons for exclusions at each stage, ideally with a flow diagram.	4
Study characteristics	18	For each study, present characteristics for which data were extracted (e.g., study size, PICOS, follow-up period) and provide the citations.	4
Risk of bias within studies	19	Present data on risk of bias of each study and, if available, any outcome level assessment (see item 12).	7
Results of individual studies	20	For all outcomes considered (benefits or harms), present, for each study: (a) simple summary data for each intervention group (b) effect estimates and confidence intervals, ideally with a forest plot.	4-6
Synthesis of results	21	Present results of each meta-analysis done, including confidence intervals and measures of consistency.	6
Risk of bias across studies	22	Present results of any assessment of risk of bias across studies (see Item 15).	8
Additional analysis	23	Give results of additional analyses, if done (e.g., sensitivity or subgroup analyses, meta-regression [see Item 16]).	7-8
**DISCUSSION**			
Summary of evidence	24	Summarize the main findings including the strength of evidence for each main outcome; consider their relevance to key groups (e.g., healthcare providers, users, and policy makers).	9
Limitations	25	Discuss limitations at study and outcome level (e.g., risk of bias), and at review-level (e.g., incomplete retrieval of identified research, reporting bias).	10
Conclusions	26	Provide a general interpretation of the results in the context of other evidence, and implications for future research.	10
**FUNDING**			
Funding	27	Describe sources of funding for the systematic review and other support (e.g., supply of data); role of funders for the systematic review.	10

From: Moher D, Liberati A, Tetzlaff J, Altman DG, The PRISMA Group (2009). Preferred Reporting Items for Systematic Reviews and Meta-Analyses: The PRISMA Statement. PLoS Med 6(7): e1000097. doi:10.1371/journal.pmed1000097

For more information, visit: **www.prisma-statement.org**.

Table S2 The results of subgroup analysis

Subgroup	No. of studies	No. of participants	No. of cases	Relative risk (95% CI[1])	I^2 (%)
**Study type**					
case control study	3	1618	974	6.59 (1.17, 37.02)	95
prospective study	16	122942	6445	1.28 (1.19, 1.38)	32
**Sample size**					
no more than 1000	5	2527	1071	3.63 (1.30, 10.15)	91
more than 1000	14	122033	6348	1.28 (1.18, 1.38)	32
**Subtype of stroke**					
ischemic stroke	7	45856	3731	2.36 (1.53, 3.63)	88
hemorrhagic stroke	4	9463	460	2.50 (0.87, 7.15)	89
**Vitamin type**					
intake	3	13432	1339	1.22 (0.96, 1.55)	53
circulating	17	111883	6150	1.93 (1.49, 2.48)	86

[1] CI, confidence interval.

Table S3 Variables adjusted in the model of each included study

Study	Variables adjusted in model
Marniemi et al, 2005	age, gender, smoking and functional capacity, for serum 25(OH)D<br>age, gender, smoking, functional capacity and weight adjusted energy intake, for vitamin intake
Bolland et al, 2010	treatment allocation (calcium or placebo) , baseline age , body weight , smoking status , systolic blood pressure , history of ischemic heart disease、stroke or transient ischemic attack , dyslipidemia, and diabetes
Drechsler et al, 2010	age, sex, atorvastatin treatment, season, coronary artery disease, congestive heart failure, systolic blood pressure, smoking, duration of dialysis, ultrafiltration volume, body mass index, levels of LDL, HDL cholesterol, C-reactive protein, HbA1c, use of beta-blockers, ACE inhibitors, diuretics,levels of parathyroid hormone, calcium, and phosphate.
Anderson et al, 2010	age, gender, hypertension, hyperlipidemia, diabetes mellitus, and peripheral vascular disease , fractures, pulmonary embolism, depression, renal failure, skeletal disorder, hypothyroidism, infection requiring medical attention, and headache
Schierbeck et al, 2012	age, smoking, blood pressure, family history of MI, education and hip/waist ratio
Kojima et al, 2012	age, total kilocalories, BMI, hypertension, diabetes mellitus, pack-years smoking, PAI, serum cholesterol, and alcohol intake
Sun et al, 2012	age at blood draw (year), menopausal status, use of postmenopausal hormone, race, smoking status ,body mass index, physical activity, use of aspirin, hypertension, high cholesterol, history of heart disease or diabetes, alcohol consumption, use of multivitamin, intakes of calcium and trans-fat, polyunsaturated fat to<br>saturated fat ratio, and glycemic load
Michos et al, 2012	age and sex,income, education, body mass index, smoking, physical activity, alcohol use, season,C-reactive protein, diabetes, hypertension, hypercholesterolemia, and race
Perna et al, 2013	age, sex, and season of blood draw, BMI, smoking, physical activity, total cholesterol, CRP, family history of CVD, fish consumption, regular multivitamin supplement intake, hypertension, DM, and CKD
Kühn et al, 2013	BMI, sex, stratified by center, age at baseline, waist circumference, alcohol intake, education level, physical activity, and smoking
Skaaby et al, 2013	study group, gender, education, season of blood sample, intake of fish, physical activity, smoking habits, body mass index, and alcohol consumption
Ford et al, 2014	age, sex, time since fracture, type of fracture, diabetic status, smoking status, and myocardial infarction

Schneider et al, 2015	age, sex, race, center, education, physical activity, smoking status, and body mass index
Judd et al, 2016	age, race, age*race interaction, sex, season of blood draw, systolic blood pressure, body mass index, diabetes, left ventricular hypertrophy, current smoking, atrial fibrillation, coronary heart disease, estimated glomerular filtration rate, log-transformed albumin to creatinine ratio and use of anti-hypertensive medications, statins, aspirin serum calcium, phosphorus, intact parathyroid hormone, high-density lipoprotein, low-density lipoprotein, total cholesterol and triglyceride concentrations
Zittermann et al, 2016	gender, body mass index, ECMO implantation, aspartate aminotransferase, white blood cells, C-reactive protein, and platelets
Alfieri et al, 2017	age, gender, body mass index levels, ethnicity, smoking status, diabetes, dyslipidemia, antihypertension, hypoglycemic, and hypolipemiant drugs
Tan et al, 2017	N.A.
Leung et al, 2017	age, sex, BMI, lifestyle factors (smoking, drinking, physical activity, education levels), biomarkers of vitamin D (season, eGFR, serum calcium, serum phosphate, serum alkaline phosphatase, serum parathyroid hormone levels), history of lipid lowering medications, and history of antihypertensive drugs
Afzal et al, 2017	age, sex, smoking status, cumulative tobacco consumption, alcohol consumption, leisure time physical activity, body mass index, income, diabetes mellitus, ratio of non–high-density lipoprotein (HDL) to HDL cholesterol, stroke in parents, atrial fbrillation, estimated glomerular fltration rate, antihypertensive medication, month and year of blood sample, and study
